# Bronchial Artery Embolization for a Mediastinal Aortopulmonary Paraganglioma Safe Resection: A Case Report

**DOI:** 10.1155/2024/5764491

**Published:** 2024-11-16

**Authors:** Diego Salcedo Miranda, Jorge Roberto Galvis O., Luis Gerardo García-Herreros, David Torres Cortes, Oscar Rivero Rapalino

**Affiliations:** ^1^Department of Thoracic Surgery, Universidad El Bosque/Instituto Nacional de Cancerologia, Bogotá, Colombia; ^2^Department of General Surgery, Universidad El Bosque, Bogotá, Colombia; ^3^Department of Thoracic Surgery, Fundación Santa Fe de Bogotá, Bogotá, Colombia; ^4^Department of Interventional Radiology, Fundación Santa Fe de Bogotá, Bogotá, Colombia

**Keywords:** embolization, mediastinal chemodectoma, sternotomy

## Abstract

Paraganglioma of the middle mediastinum has a prevalence of 1%–2% of paragangliomas and less than 1% of mediastinal masses. It is generally asymptomatic and can easily be confused with other pathologies. The following is the case of a 50-year-old patient who, as an incidental finding, documented an injury between the aorta and the pulmonary artery, hypervascularized, which was embolized prior to surgery, which facilitated the complete resection of the lesion by sternotomy. With favorable evolution of the patient and discharge on the fourth postoperative day. A thorough review of the literature on the diagnostic and treatment approach to this pathology has been also carried out.

## 1. Introduction

Mediastinal aortopulmonary paraganglioma (APPG) is a very rare pathology, with only ~150 cases reported in the literature [1], equivalent to 1%–2% of paragangliomas and less than 0.3% of mediastinal masses [1]. It is commonly confused with mediastinal lymphadenopathy. However, they have high contrast medium uptake. Among its clinical manifestations, the patient is usually asymptomatic, but they can present with nonspecific symptoms such as diaphoresis, tachycardia, headache, and arterial hypertension because of its catecholaminergic effect [2]. Its location is frequent in the middle mediastinum [3]. Given its low prevalence and the multidisciplinary approach that was performed, embolizing the lesion and subsequently surgical resection, it is important to describe the current clinical case, its indications for resection and surgical approach.

## 2. Materials and Methods

**Figure 1:**
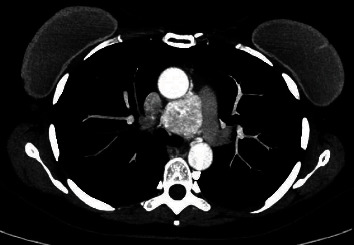
Axial section: mediastinal paraganglioma located between the aorta and the pulmonary artery with dimensions of 39 × 42 mm.

**Figure 2:**
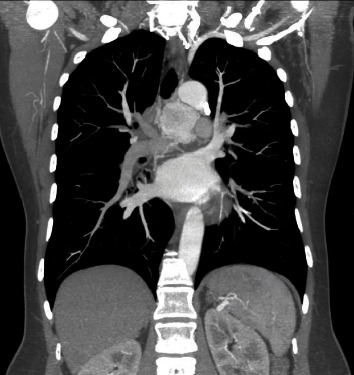
Coronal section: mediastinal paraganglioma with arterial vessel dependent on the left bronchial artery.

The case of a 50-year-old patient with no significant history is presented, in whom a mediastinal APPG, was documented as an incidental finding in a transthoracic echocardiogram. A contrast-enhanced computed tomography of the chest was performed in axial and coronal sections where a well-defined, hypervascular mediastinal mass was identified, located between the aorta and the pulmonary artery with dimensions in the axial section of 39 × 42 mm (Figures 1 and 2). In the axial section, a nutrient vessel dependent on the left bronchial artery is evident.

**Figure 3:**
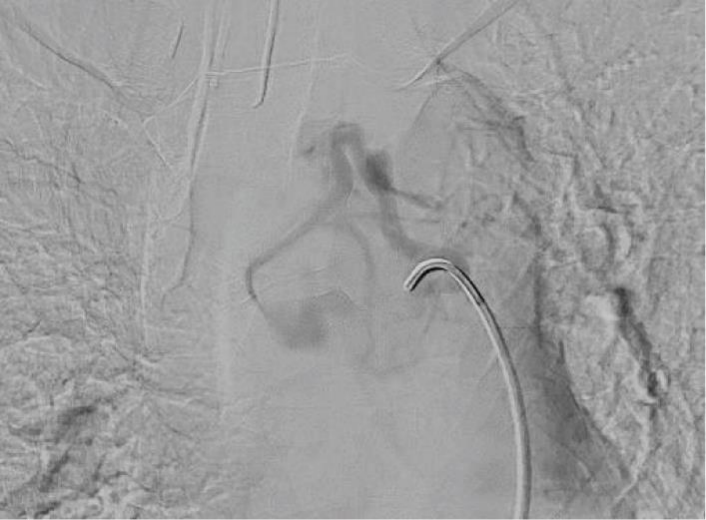
Trunk of the left bronchial artery as the main afferent of the mediastinal lesion (paraganglioma).

**Figure 4:**
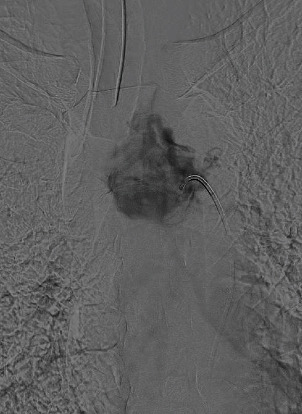
Hypervascularized mediastinal paraganglioma with contrast medium uptake in angiography.

**Figure 5:**
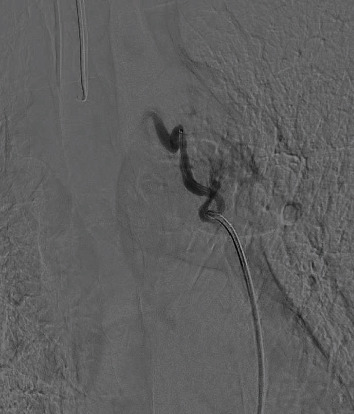
Mediastinal paraganglioma lesion, final angiographic control with digital subtraction, with adequate endovascular devascularization.

The angiographic image with digital subtraction in anteroposterior projection, the trunk of the left bronchial artery is identified as the main afferent of the already known hypervascularized mediastinal lesion, while in the late acquisitions, significant enhancement of the mass is evident. No arterial or venous afferents were documented with vascular structures of the neck and/or thorax (Figures 3–5) (videos of the procedure are available in the Supporting Information section).

In the final angiographic control with digital subtraction, adequate endovascular devascularization of the mediastinal mass is documented using microspheres with diameters between 300–500 and 500–700 μm.

### 2.1. Procedure

Procedure performed under general anesthesia. Ultrasound-guided puncture of the right common femoral artery is performed using a micropuncture set (silhouette), and after this, with an exchange technique, a short 5F vascular introducer (avanti) is positioned. Through the vascular introducer, using fluoroscopic guidance, a 0.035” hydrophilic guidewire (terumo) and a C2 curve angiographic catheter is ascended and navigated inside the right iliac artery and the aorta until it is positioned in the aortic arch. After this, the hydrophilic guide is removed, and using the catheter and the 50% diluted contrast medium, the right bronchial artery is catheterized, from which the greatest vascular contribution of the mass was documented (Video 1). Angiographic acquisitions are performed, which help in the vascular characterization by discarding communications or leaks with the spinal cord, vertebral bodies, and/or subclavian arteries.

Through the C2 catheter, a Progreat 2.8F microcatheter is coaxially ascended, advancing it until ultraselective catheterization of the arterial tributaries is achieved. With the microcatheter in position, particle administration is started (Embosphere) initially from 300–500 μm until the devascularization of most of the intraparenchymal part of the mass is achieved, then particle administration is performed (Embosphere) between 500 and 700 μm to achieve complete devascularization of the mass, the microcatheter is withdrawn and angiographic control acquisitions are performed through the C2 catheter observing adequate devascularization of the mass (Video 2).

Subsequently, the right subclavian artery and the internal mammary artery were selectively catheterized without demonstrating arterial afferent to the mediastinal lesion (Video 3).

The catheter and the vascular introducer were removed and the mechanical suture (Perclose ProGlide) was placed.

## 3. Results

**Figure 6:**
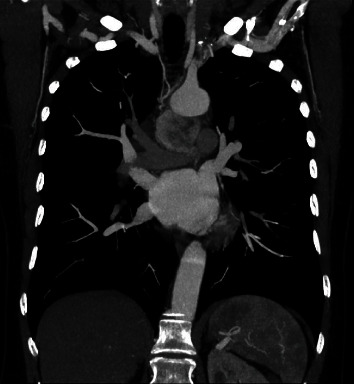
Tomographic coronal section, 27 days posterior a embolización, con visualización de áreas de necrosis central.

**Figure 7:**
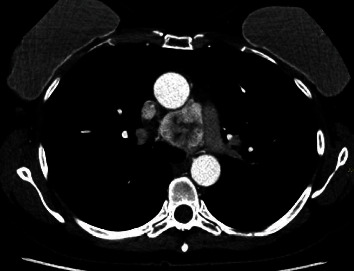
Axial section, tomographic control 27 days after embolization, with evidence of areas of central necrosis.

In the tomographic control performed 27 days after arterial embolization, a significant generalized decrease in the vascularization of the lesion was documented with some areas of central necrosis (Figures 6 and 7).

**Figure 8:**
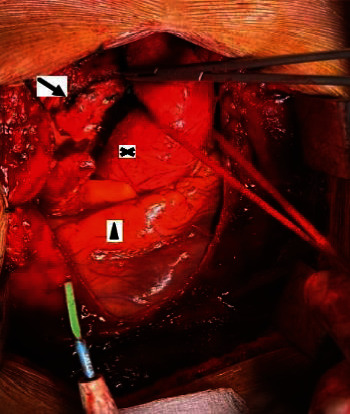
Surgical resection of mediastinal lesion (sternotomy approach), ascending aorta (equis), paraganglioma (arrow), and heart (arrowhead).

**Figure 9:**
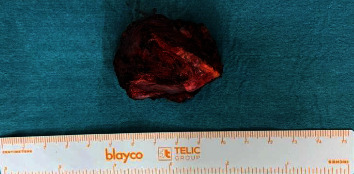
Surgical specimen: resection of a mediastinal paraganglioma by median sternotomy.

After embolization, surgical resection is carried out. The approach was performed through a median sternotomy, identifying a mass located in the lesser curvature of the aortic arch, which compromised the bifurcation of the pulmonary artery and its left and right branches, as well as the medial portion of the superior vena cava with the anterior surface of the trachea. The main artery was identified at the level of the ligamentum arteriosus, coming from the left bronchial artery, which was ligated and clipped, and a second main artery in the posterior part of the tumor, which was ligated and clipped; allowing the mass to be resected without complications, with little surgical bleeding (Figure 8) and complete extraction of the surgical piece without rupture of its capsule (Figure 9).

The final pathology report described paraganglioma-type neoplasia without extracapsular involvement.

The patient had a satisfactory clinical evaluation and on the fourth postoperative day was discharged without complications during her hospital stay. There was no evidence of paraganglioma recurrence on the third postoperative month tomographic image control.

The patient's quality of life improved considerably, and she is very satisfied with the treatment received.

The patient gives informed consent so that her case and management received are documented because it is of public interest and may help in the evaluation and management of other patients with similar pathology.

## 4. Discussion

Paragangliomas are rare benign endocrine neoplasms. Most are slowly growing and asymptomatic [1, 4]. It can be classified into APPG, which originates in the anterior or middle mediastinum, and aorticosympathetic paraganglioma (ASPG), which originates in the posterior mediastinum. APPG occurs more frequently in people over 40 years of age because it is located between the ascending aorta and the trunk of the pulmonary artery, which agrees with the patient we present in this article [5, 6].

The differential diagnosis at the time of surgery includes infection, lymphadenopathy, neoplasia, and metastasis [1].

In tomographic images, they are highly contrast-enhancing tumors. Small tumors have uniform contrast enhancement. Large tumors have heterogeneous uptake, showing necrosis. They have a salt (foci of hemorrhage) and pepper (absence of blood flow) pattern [5]. Diagnosis by mediastinoscopy is not recommended due to the high bleeding rate; the diagnosis must be imagined with pathological confirmation. Although other authors report that percutaneous biopsy guided by tomography can be performed [7].

The majority are asymptomatic tumors and do not secrete catecholamines, but they can cause chest discomfort or neurological symptoms; however, they can secrete catecholamines, causing symptoms such as diaphoresis, headache, tachycardia, and hypertension [2]. In any patient in whom a paraganglioma is suspected, a biochemical diagnosis must be made, measuring fractionated metanephrines in plasma and urine, which are the most sensitive and specific tests [8].

It is very important to treat this pathology as soon as it is diagnosed, even if it sometimes occurs in asymptomatic people as in this case, because if it is not done, the paraganglioma tends to continue growing. Reaching the point that it will compress, obstruct, and invade vital structures such as the large mediastinal vessels, lungs, heart; generating different pathologies secondary to this, which can lead to the death of the patient [9].

Regarding treatment, the cornerstone is surgical resection. Due to the proximity of this lesion to vital vascular structures, complete resection rate of the lesion is 75%, which improves considerably if the lesion can be previously embolized, making it vital to be able to perform the complete resection with safety margins of the lesion because these tumors tend to recur locally.

As demonstrated in the series by Al-Jehani et al. [9], in which in patients with complete resection the survival rate was 84.6% with an average survival time of 125 months; in contrast to patients with incomplete resection, in whom the survival rate was 50%, with an average lifespan of 71 months [9, 10].

A study published in 2021 [11, 12] shows how presurgical embolization is essential in the multidisciplinary and perioperative management of this pathology and should be performed if there is a high risk of bleeding, which can occur in large tumors or that, due to location, makes complete resection of the tumor more complex.

Rakovich et al. [13] proposed in the same way that paragangliomas of the neck and carotid body were embolized, this management could be performed with mediastinal paragangliomas.

Preoperative embolization should be performed 1–7 days before surgery [13], generating tumor cytoreduction [14], and decreasing the risk of bleeding by ~60%–70% bleeding volume [13], which is the most common cause of perioperative death. Common in these patients, which occurs in up to 7%–8%, as evidenced in the series by Brown et al. [15].

Through angiography, it is evident that branches of the right bronchial artery, right and left internal mammary artery, left thyrocervical trunk, and aberrant left bronchial artery are the ones that most frequently irrigate these mediastinal paragangliomas. The embolization of all these branches generates the almost complete disappearance of the tumor blush, which in control angiography can show that these branches that supplied the tumor have disappeared. Generating great benefits in patient management, with very low complication rates derived from the procedure [13].

There may be complications following embolization. The most common is pleuritic pain, it appears in the first 24 h and is managed with analgesics [16]. Device migration may occur, it is the most feared complication because it can cause stroke, it occurs in 3% of the cases; percutaneous puncture is attempted to deflate and remove the device [16]. There is also risk of spinal cord ischemia secondary to inadvertent embolization of the anterior or posterior spinal arteries via radiculomedullary arteries that can originate from the bronchial arteries is very serious, occurring in 1%–6% of cases [16].

Another complication is hypertensive crisis, which sometimes occurs when the tumors are endocrinologically functional. Therefore, it is important to functionally evaluate with catecholamine secretion tests, prior to embolization [17].

Another measure that can be taken to reduce complications in the management of this pathology is to administer medical treatment with alpha and/or beta receptor blockers 7–10 days before surgery, which reduces complications induced by catecholamines, for example, perioperative hypertensive crisis, which is one of the two greatest risks of surgery and embolization, along with to perioperative bleeding [2], reducing these risks from 69% to 4% [8, 15, 18].

Another type of surgical approach that can be performed in this pathology is with a minimally invasive technique, whether assisted by a robot or by thoracoscopy, depending on the experience of the surgical center and the supplies available, keeping in mind that there is the possibility of converting to open surgery in up to 30% of cases as mentioned in Dr Senthilkumar's series [17]

If the lesion is adhered to the cardiac chambers, partial attriectomies or other procedures may be required, prior cardiopulmonary bypass, to guarantee a better survival rate that is directly proportional to complete surgical resection without generating lesions [19].

It is important to mention that in other types of mediastinal hypervascular masses, such as cavernous mediastinal hemangioma; which is a benign mediastinal tumor, very rare and 50% of the time it occurs asymptomatically [20]. Mediastinal glomangioma which is more common inmen and asymptomatic most of the time [21]. A presurgical embolization approach can be performed to facilitate complete surgical resection and reduce the probability of bleeding [20, 21].

In the postoperative follow-up of the patient, it is recommended to perform a genetic study, given that up to 25% of cases are hereditary [1, 8] and annual metanephrine analysis to detect local or metastatic recurrence, which occurs in up to 17% to 20% [22] or much higher, reaching up to 50% with the capacity to metastasize at 26%, as shown in their article by Lamy et al. [12]. It is suggested that follow-up should be carried out for at least 10 years, while other authors such as Brown et al. in their experience at Mayo Clinic express that follow-up should be lifelong [11, 15].

The advance in diagnostic and therapeutic imaging studies is very important for the prompt and adequate diagnosis of this pathology, detecting it in early stages, since it can be confused with other pathologies and be taken to biopsy with risk of significant bleeding. Also given the advance in embolization techniques, it becomes an increasingly safe and effective procedure for the management of this type of lesions.

## 5. Conclusions

• Mediastinal paraganglioma is a rare pathology that can be confused with other more prevalent pathologies.
• The approach must be multidisciplinary, in which, prior to surgical resection, embolization of the lesion is suggested to reduce the risk of intraoperative bleeding and medical management with alpha and beta blockers to reduce the risk of perioperative hypertensive crisis.
• Complete surgical resection is essential given the survival rate over time. It will depend on this.
• Postoperative follow-up of the patient must continue over time, to rule out recurrence of the disease.
• Presurgical embolization may be used in the management of mediastinal paraganglioma and other mediastinal lesions

## Data Availability

The data that support the findings of this study are available from the corresponding author upon reasonable request.

## Supporting Information

Supporting Information Video 1. Angiographic acquisitions. Video 2. Embolization and adequate devascularization of the mass. Video 3. The right subclavian artery and the internal mammary artery were selectively catheterized, without demonstrating arterial afferent to the mediastinal lesion.
