# Supplementary material for: Bronchial Artery Embolization for a Mediastinal Aortopulmonary Paraganglioma Safe Resection: A Case Report
Source: Case Rep Surg. 2024 Nov 16;2024:5764491. doi: 10.1155/2024/5764491 (PMC11585371; doi:10.1155/2024/5764491)
Supplement: Supporting Information — Video 1. Angiographic acquisitions. Video 2. Embolization and adequate devascularization of the mass. Video 3. The right subclavian artery and the internal mammary artery were selectively catheterized, without demonstrating arterial afferent to the mediastinal lesion. [file 5764491.f1.docx]

<https://drive.google.com/drive/folders/1QbD7WGJqTm7iLVwZ-iU4CeGI38B5mmlk>
